# Orally administration of cerium oxide nanozyme for computed tomography imaging and anti-inflammatory/anti-fibrotic therapy of inflammatory bowel disease

**DOI:** 10.1186/s12951-023-01770-0

**Published:** 2023-01-19

**Authors:** Yameng Cao, Kai Cheng, Mei Yang, Zhichao Deng, Yana Ma, Xiangji Yan, Yuanyuan Zhang, Zhenzhen Jia, Jun Wang, Kangsheng Tu, Jie Liang, Mingzhen Zhang

**Affiliations:** 1 Department of Hepatobiliary Surgery, The First Affiliated Hospital of Xian Jiaotong University, Xi’an, 710061 Shaanxi China; 2 School of Basic Medical Sciences, Xian Key Laboratory of Immune Related Diseases, Xian Jiaotong University, Xi’an, 710061 Shaanxi China; 3 Britton Chance Center for Biomedical Photonics at Wuhan National Laboratory for Optoelectronics - Hubei Bioinformatics & Molecular Imaging Key Laboratory, Department of Biomedical Engineering, College of Life Science and Technology, Huazhong University of Science and Technology, Wuhan, 430074 Hubei China; 4 Department of Emergency and Critical Care Medicine, The First Affiliated Hospital of Xi’an Jiaotong University, Xi’an, 710061 China; 5 Key Laboratory of Environment and Genes Related to Diseases, Xian Jiaotong University, Ministry of Education, Xi’an, 710061 Shaanxi China; 6 Xijing Hospital of Digestive Diseases, Air Force Military Medical University, Xi’an, 710068 Shaanxi China

**Keywords:** Inflammatory bowel disease, Cerium oxide nanozyme, CT imaging, Inflammation, Intestinal fibrosis

## Abstract

**Background:**

Inflammatory bowel disease (IBD) is a chronic nonspecific disease with unknown etiology. Currently, the anti-inflammatory therapeutic approaches have achieved a certain extent of effects in terms of inflammation alleviation. Still, the final pathological outcome of intestinal fibrosis has not been effectively improved yet.

**Results:**

In this study, dextran-coated cerium oxide (D-CeO_2_) nanozyme with superoxide dismutase (SOD) and catalase (CAT) activities was synthesized by chemical precipitation. Our results showed that D-CeO_2_ could efficiently scavenge reactive oxide species (ROS) as well as downregulate the pro-inflammatory cytokines (IL-1β, IL-6, TNF-α, and iNOS) to protect cells from H_2_O_2_-induced oxidative damage. Moreover, D-CeO_2_ could suppress the expression of fibrosis-related gene levels, such as α-SMA, and Collagen 1/3, demonstrating the anti-fibrotic effect. In both TBNS- and DSS-induced colitis models, oral administration of D-CeO_2_ in chitosan/alginate hydrogel alleviated intestinal inflammation, reduced colonic damage by scavenging ROS, and decreased inflammatory factor levels. Notably, our findings also suggested that D-CeO_2_ reduced fibrosis-related cytokine levels, predicting a contribution to alleviating colonic fibrosis. Meanwhile, D-CeO_2_ could also be employed as a CT contrast agent for noninvasive gastrointestinal tract (GIT) imaging.

**Conclusion:**

We introduced cerium oxide nanozyme as a novel therapeutic approach with computed tomography (CT)-guided anti-inflammatory and anti-fibrotic therapy for the management of IBD. Collectively, without appreciable systemic toxicity, D-CeO_2_ held the promise of integrated applications for diagnosis and therapy, pioneering the exploration of nanozymes with ROS scavenging capacity in the anti-fibrotic treatment of IBD.

**Supplementary Information:**

The online version contains supplementary material available at 10.1186/s12951-023-01770-0.

## Background

Inflammatory bowel disease (IBD) is a chronic inflammatory bowel disease that includes ulcerative colitis (UC) and Crohn's disease (CD). The pathogenesis of IBD is unclear and may be attributed to the interference of many factors, including environment, genetics, gastrointestinal dysbiosis, and infection [1, 2]. Although drug-based therapy can control inflammation to some extent, it is almost impossible to cure it completely [3]. Intestinal fibrosis is a known complication of IBD and can be present in both UC and CD, but is more common in CD. It is a chronic progressive process characterized as a dynamic multifactorial accumulation of extracellular matrix caused by a chronic inflammatory response of the intestinal tissues [4–6]. Eventually, it often develops into intestinal obstruction, a critical reason clinical patients require repeated surgery [7, 8]. However, commonly used anti-inflammatory therapies are invalid in preventing and reversing established intestinal fibrosis and obstruction [9]. Therefore, in addition to relieving the inflammatory state of the intestine, reducing the occurrence of fibrosis is also of great importance for IBD treatment.

There is growing evidence that ROS plays a crucial role in the persistence of IBD, and the removal of ROS from inflammatory sites has been demonstrated to be an effective strategy to alleviate the inflammatory state of IBD [10–13]. Interestingly, it has been reported that ROS also plays a role in regulating the process of fibrosis [14, 15]. Various factors, including cytokines, chemokines, and other molecules, can control the progression of fibrosis. Of the overall pro-fibrotic molecules, TGF-β plays a vital role [16, 17]. There are three subtypes of TGF-β in mammals: TGF-β1, TGF-β2, and TGF-β3. TGF-β1 is among the most abundant in the mammalian intestine, and its role in intestinal immunity has been extensively studied [18, 19]. As an essential regulator of fibroblast phenotype and function, TGF-β1 can induce epithelial-mesenchymal transition (EMT) through various pathways, allowing fibroblasts to be activated and transformed into myofibroblasts. Myofibroblasts are the essential effector cells responsible for the onset and development of fibrosis [20, 21]. In contrast, ROS can influence intestinal fibrosis by regulating TGF-β and its downstream pathways [14, 22]. Therefore, we hypothesized that eliminating ROS could alleviate the inflammatory state as well as the colonic fibrosis in IBD.

Recently, cerium oxide (CeO_2_) nanoparticles as nanozymes have been widely used in the therapeutic research of inflammatory diseases [23–28]. The presence of both Ce^3+^ and Ce^4+^ sites on the surface of CeO_2_ nanoparticles endows them with superoxide dismutase (SOD) and catalase (CAT) activities [29, 30]. They can effectively reduce oxidative stress levels by scavenging superoxide anion (·O_2_^−^) and hydrogen peroxide (H_2_O_2_) produced at the site of inflammation. Compared with natural enzymes, nanozymes have better stability, versatility, and recyclability [31]. In addition to possessing the activity of mimicking enzymes, CeO_2_ has been demonstrated as a Computed Tomography (CT) contrast agent for imaging gastrointestinal inflammation. Moreover, its enzymatic activity can effectively reduce the free radicals generated by X-ray radiation and mitigate the damage to the inflammation site compared with traditional contrast agents [32].

**Fig. 1:**
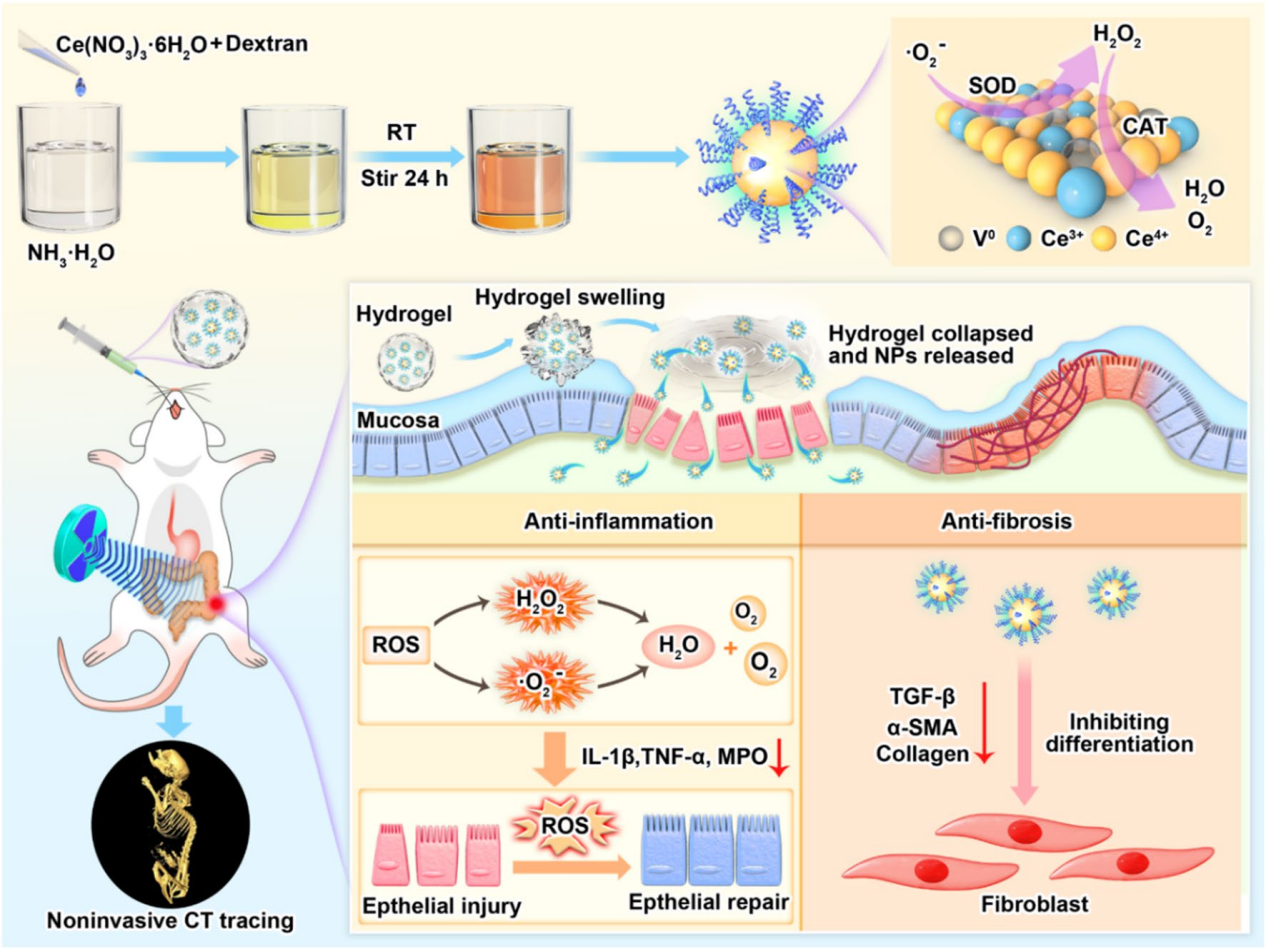
Schematic representation of the preparation of D-CeO_2_ and its anti-inflammatory/anti-fibrotic mechanism. D-CeO_2_ was synthesized by the precipitation method, and the color of the solution changed from yellow to dark brown during the synthetic process. Orally administered of D-CeO_2_ to mice by chitosan/alginate hydrogel. In the site of colitis, the hydrogel collapsed to release D-CeO_2_. On the one hand, D-CeO_2_ played a role in promoting the repair of damaged tissues by scavenging ROS. On the other hand, it alleviated fibrosis by decreasing the levels of fibrosis-related cytokines

In this study, dextran-coated cerium oxide (D-CeO_2_) nanozyme with superoxide dismutase (SOD) and catalase (CAT) activities was synthesized by chemical precipitation. D-CeO_2_ could efficiently scavenge reactive oxide species (ROS) as well as downregulate the pro-inflammatory cytokines (IL-1β, IL-6, TNF-α, and iNOS) to protect cells from H_2_O_2_-induced oxidative damage. Moreover, D-CeO_2_ could suppress the expression of fibrosis-related cytokine levels, such as α-SMA, and Collagen 1/3, demonstrating the anti-fibrotic effect. In colitis models, oral administration of D-CeO_2_ in chitosan/alginate hydrogel showed a significant therapeutic effect on colitis with anti-inflammatory and anti-fibrosis functions. D-CeO_2_ could also be employed as a CT contrast agent for noninvasive gastrointestinal tract (GIT) imaging (Fig. 1). D-CeO_2_ had excellent CT imaging and anti-inflammatory/anti-fibrotic ability in IBD therapy, which holds the prospect of application for diagnosis and treatment integration.

## Results

### Synthesis and characterization of CeO_2_ and D-CeO_2_

**Fig. 2:**
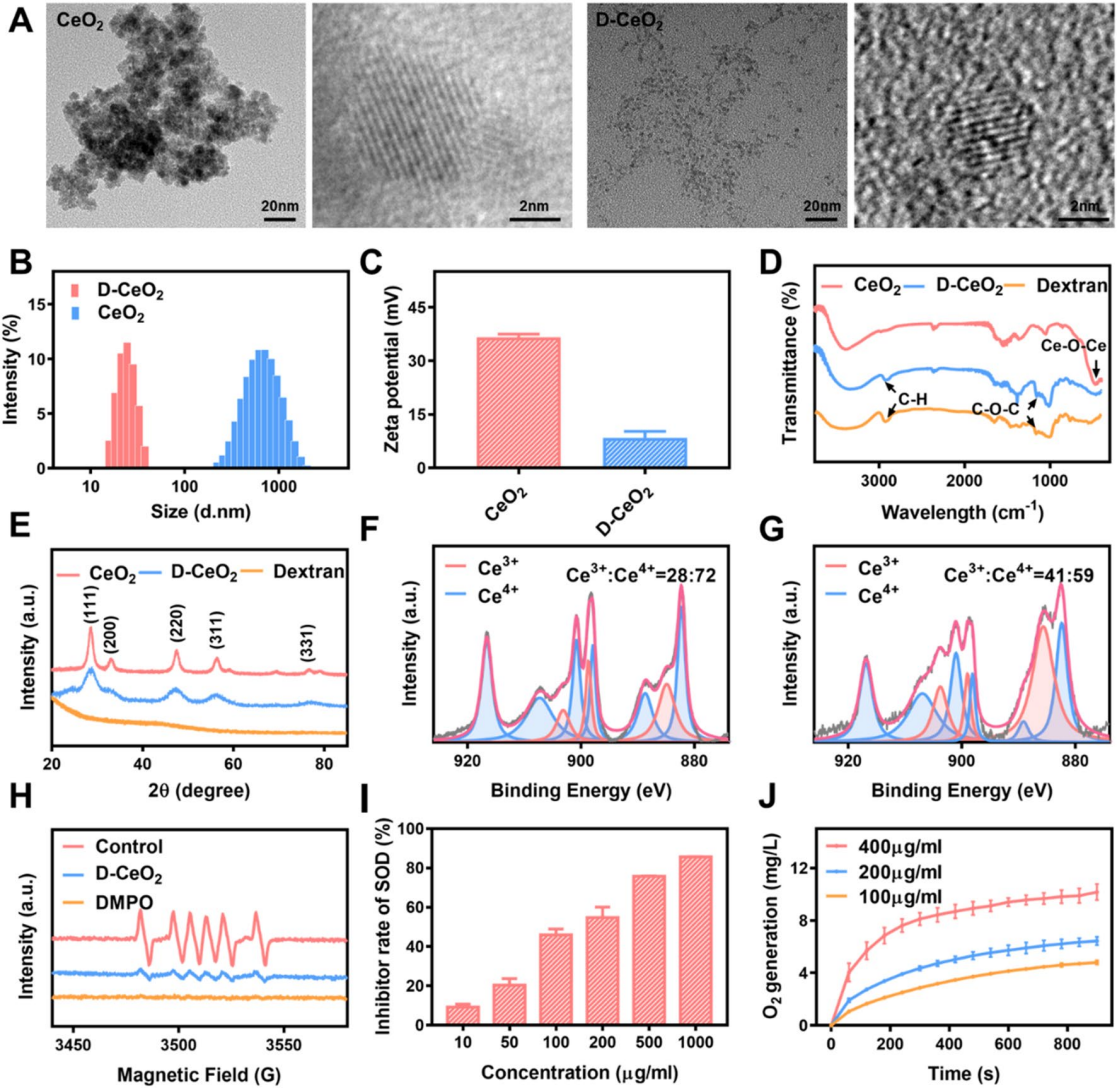
Characterization of nanoparticles. **A** Transmission electron microscopy (TEM), **B** hydrated particle size, and **C** zeta potential of CeO_2_ and D-CeO_2_. **D** FT-IR spectra. **E** XRD patterns. Analysis of the Ce^3+^/Ce^4+^ ratio in **G** CeO_2_ and **F** D-CeO_2_ by XPS. Superoxide anion scavenging by SOD-mimicking ability in different concentrations of D-CeO_2_ was analyzed using **H** ESR and **I** NBT methods. **J** Oxygen generation from H_2_O_2_ (120 mM) catalyzed by CAT-mimicking activity of D-CeO_2_

CeO_2_ and D-CeO_2_ were synthesized by the precipitation method. Transmission electron microscopy (TEM) results showed that D-CeO_2_ has a smaller particle size than CeO_2_ under the same synthesis conditions (Fig. 2A). This phenomenon should attribute to the fact that during the formation of cerium oxide nanocrystals, dextran molecules wrap around the surface of nanoparticles, preventing their further growth [33]. Dynamic light scattering (DLS) studies revealed that the average hydration particle sizes of CeO_2_ and D-CeO_2_ were about 450 nm and 30 nm (Fig. 2B). At the same time, their zeta potentials were about + 36.6 mV and + 8.4 mV, respectively (Fig. 2C). Fourier transform infrared spectroscopy (FT-IR) analysis confirmed the successful coating of dextran for CeO_2_. The characteristic peaks of Ce–O–Ce were observed at around 470 cm^−1^ for both CeO_2_ and D-CeO_2_. Notably, the characteristic peaks at 2924 cm^−1^ and 1159 cm^−1^ in the D-CeO_2_ spectrum represent the C–H bonds stretching and C–O–C vibrations, respectively, which are well fit with those in free dextran spectrum (Fig. 2D). The crystalline features of the nanoparticles were characterized by X-ray diffraction (XRD). As described in Fig. 2E, the characteristic peaks of ceria were observed at 2θ = 28.6°, 33.1°, 47.5°, 56.4° and 76.80° for CeO_2_ and D-CeO_2_. UV−vis spectrum of D-CeO_2_ indicated a maximum absorption at 290 nm (Additional file 1: Fig. S1), which is consistent with the results of previous studies [31]. All of these results indicated that CeO_2_ and D-CeO_2_ were successfully synthesized. Since the enzyme-like activity of cerium dioxide depends on the ratio of Ce^3+^ to Ce^4+^ on its surface, we quantified this ratio by X-ray photoelectron spectroscopy (XPS). As shown in Fig. 2F, G, the Ce^3+^ fraction of D-CeO_2_ (41%) is higher than that of CeO_2_ (28%). This can be explained by the fact that D-CeO_2_ has a smaller particle size than CeO_2_, and the smaller the particle size, the larger the specific surface area, and the more oxygen defects, the larger the Ce^3+^/Ce^4+^ values, thus significantly enhancing the ROS scavenging capacity [34, 35].

Next, we evaluated the mimetic enzyme activities of D-CeO_2_, including SOD-mimicking and CAT-mimicking activity. ·O_2_^−^ can be trapped DMPO to form the spin adduct DMPO/·O_2_^−^. The SOD-mimicking activity was evaluated by detecting the DMPO/·O_2_^−^ signal using electron spin resonance spectroscopy (ESR). Notably, the DMPO/·O_2_^−^ signal decreased sharply after adding 100 μg/mL D-CeO_2_, demonstrating the ability of D-CeO_2_ to scavenge ·O_2_^−^ (Fig. 2H). The results of the NBT method further validated this conclusion, and with increasing concentration, it exhibited an enhanced ·O_2_^−^ scavenging ability (Fig. 2I). H_2_O_2_ is generated by ·O_2_^−^ disproportionation, which can be further decomposed into H_2_O and O_2_ by CAT enzyme activity. Using a dissolved oxygen meter, the CAT-mimicking activity of D-CeO_2_ was investigated by monitoring the catalytic decomposition of O_2_ generated by H_2_O_2_. As shown in Fig. 2J, D-CeO_2_ could catalyze the production of O_2_. In addition, as the concentration increased, D-CeO_2_ showed enhanced H_2_O_2_ scavenging ability. Compared with standard SOD and CAT, the activity of 100 µg/mL D-CeO_2_ was close to that of 0.1 U/mL SOD and 0.02 U/mL CAT (Additional file 1: Fig. S2A, B).

The complex gastrointestinal environment poses many significant problems to the orally administered of nanomedicines [36–38]. To study the ROS scavenging ability of D-CeO_2_ in the gastrointestinal tract, D-CeO_2_ was treated at different temperatures (including 4, 25, and 37 °C) and different pH solutions (including 1.5, 6.0, 7.4, and 8.0) for 4 h, corresponding to the maximum time of gastric transit in humans [38]. Compared with equal concentrations of untreated D-CeO_2_, the ROS scavenging ability of D-CeO_2_ was essentially unchanged, except for a slight decrease in SOD-mimicking activity after treatment with pH 1.5 solution (Additional file 1: Fig. S2C, D). This may be attributed to the disruption of the dextran envelope after acidic treatment, which future affected the detection of SOD-mimicking activity. To evaluate the stability of D-CeO_2_, particle sizes at different temperature and GIT pH conditions were tested and compared, As shown in Additional file 1: Fig. S2E, F, the size did not affect by temperature and could be changed according to the pH values, indicating the stability of D-CeO_2_ under GIT conditions. Therefore, we chose hydrogels to deliver the D-CeO_2_ from damage by gastric acid to protect its structure because hydrogels could reduce the contact of nanoparticles with gastric acid medium [39]. As shown in Additional file 1: Fig. S3A, in the acidic pH environment, the hydrogel was gel-like and wrapped the D-CeO_2_ well. While in alkaline pH environment, the hydrogel collapsed into white flocs and the D-CeO_2_ were released. Next, we investigated D-CeO_2_ release in different pH environments, as shown in Additional file 1: Fig. S3B, in the acidic pH environment, D-CeO_2_ release was low, while in the alkaline pH environment, D-CeO_2_ release could reach 53% at 24 h. Next, we investigated the enzyme activity of D-CeO_2_ after their release from the hydrogel. As shown in Additional file 1: Fig. S3C, D, at 24 h, with 53% release efficiency, D-CeO_2_ still kept higher SOD mimetic-, and CAT mimetic- enzyme activities, indicating the robust ROS-scavenging ability.

### In vitro ROS scavenging and anti-inflammatory effects of D-CeO_2_

**Fig. 3:**
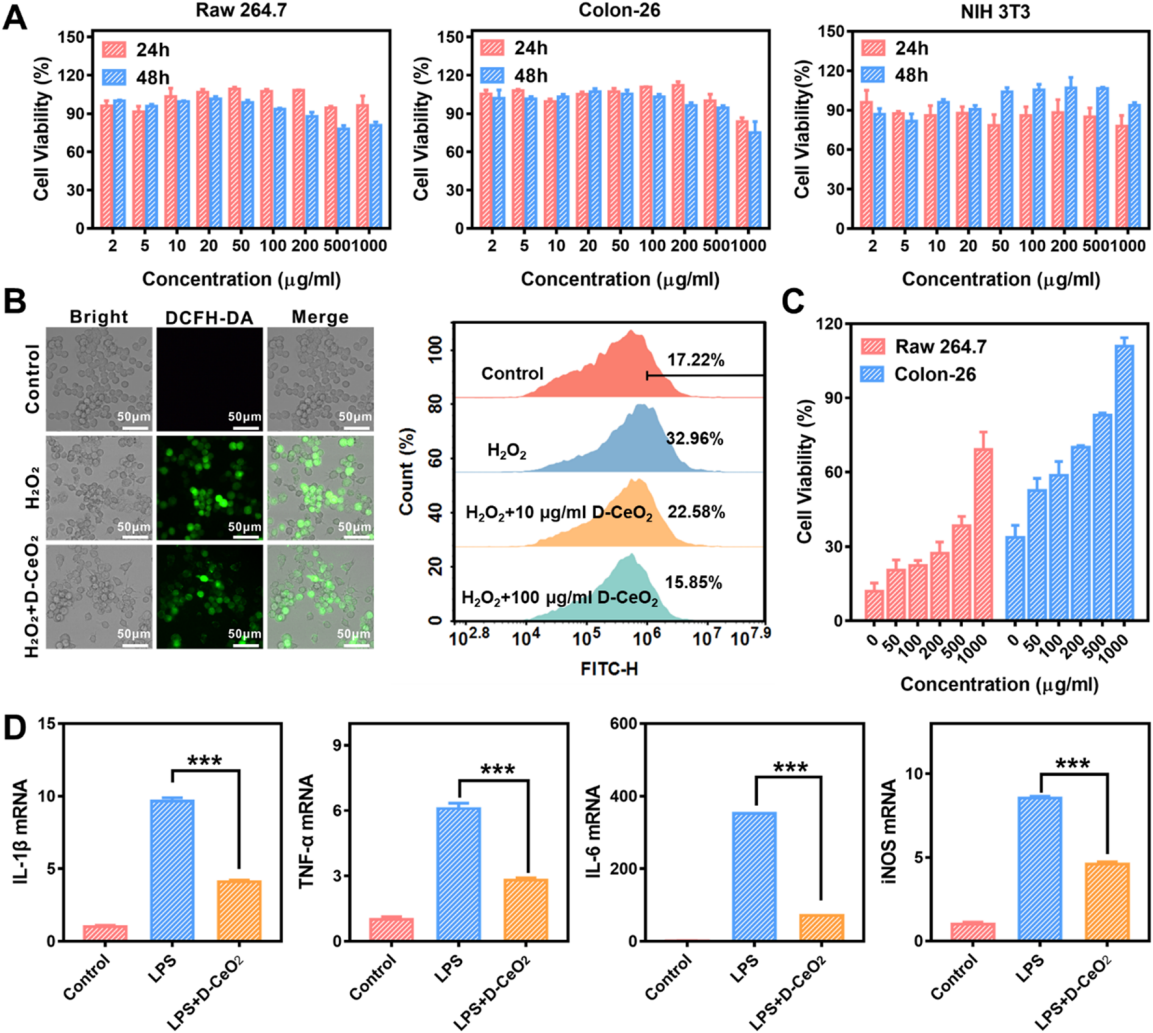
ROS scavenging and anti-inflammatory effects of D-CeO_2_. **A** Raw 264.7, Colon-26, and NIH 3T3 cell viability after incubation with D-CeO_2_ for 24 h and 48 h, respectively. **B** ROS scavenging activities in H_2_O_2_ stimulated Raw 264.7 cells were investigated by evaluating the fluorescence of DCFH-DA and flow cytometry. **C** Protective effect of D-CeO_2_ against H_2_O_2_ induced oxidative damage in Raw 264.7 and Colon-26 cells. **D** Relative mRNA levels of IL-1β, TNF-α, IL-6, and iNOS in LPS-induced Raw 264.7 cells after indicated treatments. One-way of ANOVA were performed for statistical comparison, *p < 0.05, **p < 0.01, ***p < 0.001

To evaluate the ROS scavenging and anti-inflammatory effects of D-CeO_2_, the internalization of D-CeO_2_ by cells was first investigated. All the cell lines (Raw 264.7 macrophages, Colon-26 cells, and NIH 3T3 cells) showed efficient internalization of D-CeO_2_ by observing the fluorescence of DiL inside the cells (Additional file 1: Fig. S3). The in vitro biocompatibility was then evaluated by MTT assay. As shown in Fig. 3A, D-CeO_2_ had undetectable toxicity to these three cell lines at a concentration of up to 1 mg/mL. Calcein-AM/PI co-staining further verified the great biocompatibility (Additional file 1: Fig. S4). Next, we established an intracellular inflammation model by treating cells with H_2_O_2_ to demonstrate whether D-CeO_2_ could protect cells from ROS-induced damage. After H_2_O_2_ treatment, cells were stained with the ROS-sensitive fluorescent DCFH-DA. Then intracellular ROS levels were detected by fluorescence microscopy and quantified by flow cytometry. Satisfactorily, D-CeO_2_ treatment could reduce intracellular ROS levels significantly compared to H_2_O_2_-treated only (Fig. 3B). In addition, we used H_2_O_2_ as an oxidative stress inducer to explore the protective effect of D-CeO_2_ on cells. As shown in Fig. 3C, cell viability was significantly decreased after H_2_O_2_ treatment while gradually recovering with increasing concentrations of D-CeO_2_ treatment, indicating that D-CeO_2_ could protect cells from H_2_O_2_-induced oxidative damage.

To further understand the anti-inflammatory capacity of D-CeO_2_, we evaluated its effect on the levels of several key pro-inflammatory cytokines. LPS treatment could result in the upregulation of IL-1β, IL-6, TNF-α, and iNOS pro-inflammatory cytokine levels. However, the levels of all these pro-inflammatory cytokines were decreased in D-CeO_2_ pretreated Raw 264.7 cells (Fig. 3D), demonstrating the significant anti-inflammatory ability of D-CeO_2_.

### In vitro anti-fibrotic effects of D-CeO_2_

**Fig. 4:**
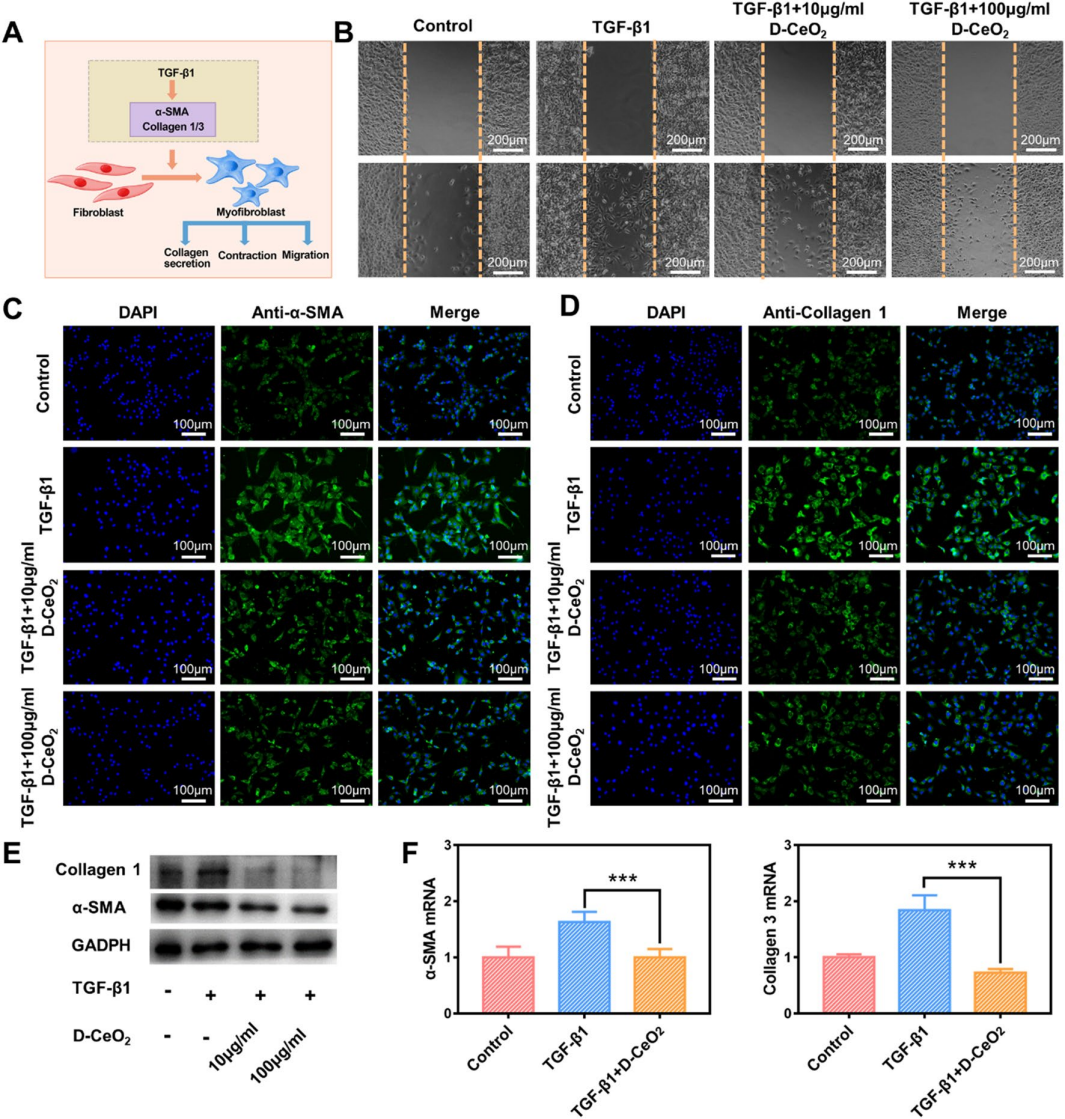
Anti-fibrotic effects of D-CeO_2_. **A** Schematic diagram of TGF-β1-induced fibrosis. **B** Scratch test in TGF-β1-induced NIH 3T3 cells after indicated treatments. Immunofluorescence of **C** α-SMA and **D** Collagen 1. **E** Western Blot. **F** Relative mRNA levels of α-SMA, Collagen 3 in TGF-β1-induced NIH 3T3 cells after indicated treatments. One-way of ANOVA were performed for statistical comparison, *p < 0.05, **p < 0.01, ***p < 0.001

The excessive accumulation of myofibroblasts is closely related to the development of fibrosis. In fibrotic diseases, major marks of myofibroblast differentiation include overexpression of α-smooth muscle actin (α-SMA), enhanced cell proliferation, and overproduction of collagen (Fig. 4A) [40, 41]. TGF-β is a central regulator that activates fibroblasts and induces fibrosis, in which TGF-β1 plays a critical role [4]. The differentiation of fibroblasts to myofibroblasts can occur through TGF-β-related signaling pathways [42, 43]. Therefore, we selected NIH 3T3 cells to establish a cellular model of fibrosis using TGF-β1 as an inducer to perform D-CeO_2_ anti-fibrosis studies.

Scratch healing of NIH 3T3 cells was significantly accelerated after a 24 h application of 10 ng/mL TGF-β1, while slowed down by co-treatment with TGF-β1 and D-CeO_2_ (Fig. 4B). Immunofluorescence staining revealed that the expression of α-SMA and Collagen 1 was upregulated in NIH 3T3 cells after TGF-β1 application. In contrast, their expression was significantly decreased in cells pretreated with D-CeO_2_ (Fig. 4C, D), suggesting that D-CeO_2_ treatment successfully reversed TGF-β1-induced fibroblast activation. The quantification of fluorescence intensity was shown in Additional file 1: Fig. S5A, B. Western blot also verified this conclusion (Fig. 4E). In addition, we also studied the influence of D-CeO_2_ on the mRNA levels of fibrosis-related genes by qRT-PCR. As with previous results, D-CeO_2_ pretreatment similarly downregulated the mRNA levels of these genes (α-SMA, and Collagen 3) (Fig. 4F), further demonstrating the anti-fibrotic effect of D-CeO_2_.

### The CT imaging property of D-CeO_2_

**Fig. 5:**
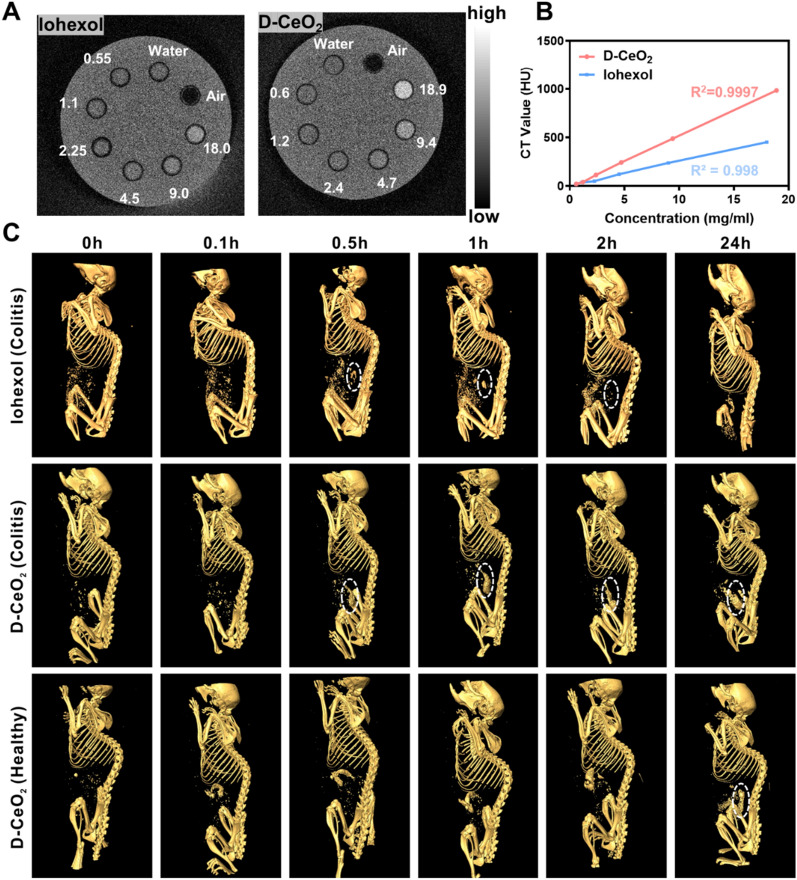
CT imaging of D-CeO_2_. **A** In vitro CT imaging of iohexol (0.55, 1.1, 2.25, 4.5, 9.0, 18.0 mg/mL) or D-CeO_2_ (0.6, 1.2, 2.4, 4.7, 9.4, 18.9 mg/mL) at different concentrations. **B** Corresponding CT values. **C** Gastrointestinal tract imaging in colitis or healthy mice after oral administration

Iohexol was used as a control to investigate the CT imaging property of D-CeO_2_. Firstly, we quantified the Ce content in D-CeO_2_ by ICP-MS and calculated that the CeO_2_ content was 12.4%. Since CeO_2_ is the central part that acts as the contrast agent in D-CeO_2_, we used the CeO_2_ concentration as the standard for comparison with iohexol. As depicted in Fig. 5A, the CT imaging ability of iohexol and D-CeO_2_ were both enhanced with increasing concentrations. The statistical results showed a linear relationship and slightly higher CT values for D-CeO_2_ at the same concentration compared to iohexol (Fig. 5B), which indicated the feasibility of D-CeO_2_ for CT monitoring at the animal level.

To prove this hypothesis, DSS-induced acute colitis mice were given iohexol and D-CeO_2_ orally and CT imaging was performed at scheduled time intervals (0, 0.1, 0.5, 1, 2, 24 h). It could be observed that iohexol started to be enriched in the gastrointestinal region 30 min after feeding, approaching its peak at 1 h, and almost completely metabolized from the gastrointestinal tract starting at 2 h. In contrast, the gastrointestinal tract of mice was visible 30 min after oral administration of D-CeO_2_ and had been maintained in the substantial signal range since then. This suggests that D-CeO_2_ has superior CT imaging ability than iohexol. In addition, the enrichment of D-CeO_2_ in the colonic fraction was only observed at 24 h in healthy mice, while enriched more quickly in colitis mice (Fig. 5C, Additional file 1: Fig. S7). This could be attributed to the high affinity of dextran for the inflammatory site. During gastrointestinal transport, the negatively charged intestinal mucus layer produced by goblet cell is able to trap nanocarriers with surface properties that are strongly positively charged and hydrophobic. Therefore, delivery systems with electrically neutral and hydrophilic surfaces are ideal for mucus permeation. As a natural polysaccharide, dextran itself has the advantages of good biocompatibility and non-immunogenicity, the neutral charge of dextran can facilitate the delivery effect [44]. More importantly, since dextran can bind to scavenger receptors on macrophages at the site of inflammation, thus exhibiting a high affinity and aggregation to sites of colonic inflammation [45].

### In vivo biocompatibility of D-CeO_2_

**Fig. 6:**
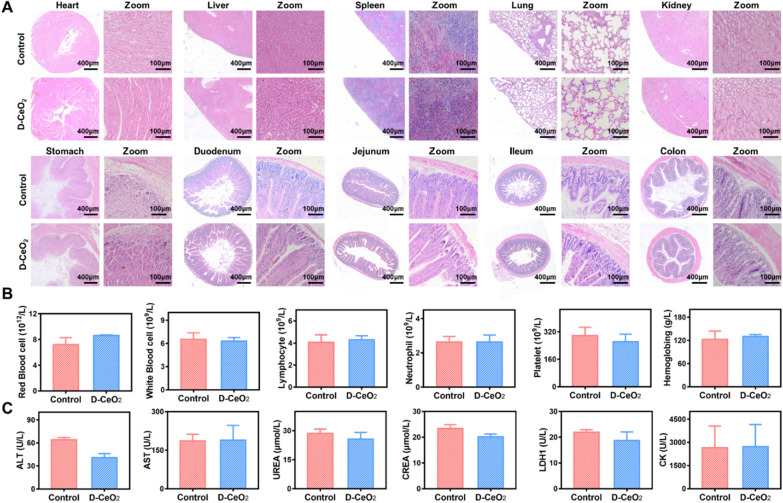
In vivo biocompatibility evaluation. **A** H&E staining, **B** blood routine index tests, and **C** blood biochemistry. T-tests were performed for statistical comparison, *p < 0.05, **p < 0.01, ***p < 0.001

Biocompatibility plays a vital role in the potential clinical application of nanomaterials, and the in vivo biocompatibility of D-CeO_2_ was systematically evaluated. C57BL/6 mice were continuously orally administered D-CeO_2_ (30 mg/kg) for 7d and sacrificed on the 30th day. The vital tissues and blood were removed for analysis. Histological evaluation of vital organs was performed by H&E staining. As presented in Fig. 6A, there were no significant histological changes and cell destruction in D-CeO_2_-treated mice compared to healthy mice. In addition, the blood routine index, including red blood cell count, white blood cell count, lymphocyte count, neutrophil count, platelet count, and hemoglobin, exhibited no significant difference in the D-CeO_2_ treated group (Fig. 6B). Typical biochemical indicators, including alanine aminotransferase (ALT), aspartate aminotransferase (AST), urease (URE), creatinine (CREA), lactate dehydrogenase (LDH1) and creatine kinase (CK), were within normal values or not significantly different (Fig. 6C). These findings confirmed that D-CeO_2_ had no significant adverse effects, promising the potential for clinical application.

Next, we investigated the biodistribution of DIR-labeled D-CeO_2_ nanoparticles in normal mice and DSS-induced mice, respectively. As shown in the Additional file 1: Fig. S8A, after 12 h of oral administration, fluorescent signals were observed only in the segments of the gastrointestinal tract, while no accumulation was observed in the vital organs such as heart, liver, spleen, lung and kidney. Interestingly, a more pronounced fluorescence signal was observed in the colitis mice compared to normal mice, further demonstrating that the modification of dextran makes it targeted. The fluorescence intensity statistics of the colon and vital organs were shown in Additional file 1: Fig. S8B, C.

### The therapeutic effect of D-CeO_2_ on TNBS-induced colitis

**Fig. 7:**
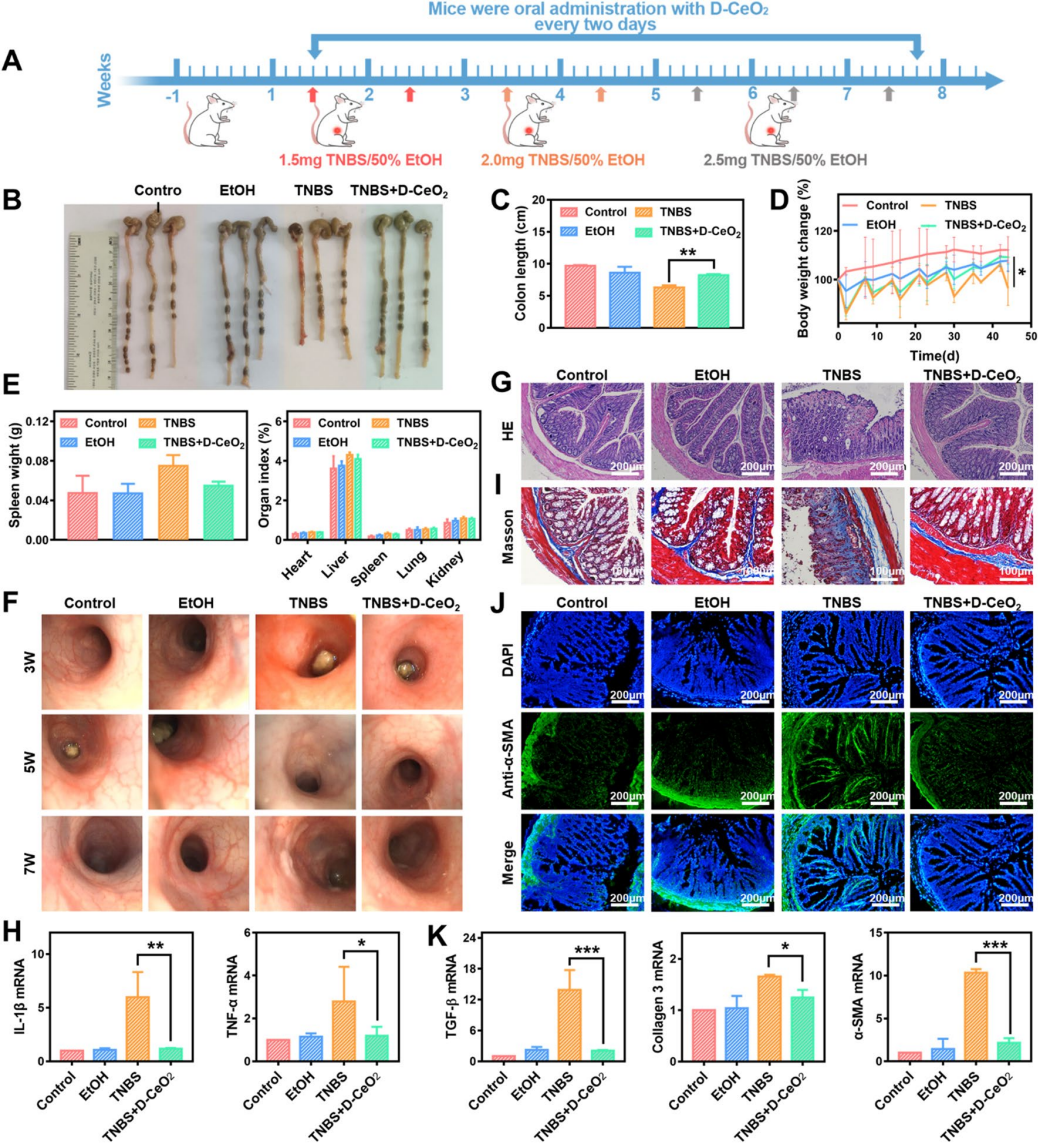
The anti-inflammatory and anti-fibrotic effects of D-CeO_2_ in TNBS-induced colitis. **A** The scheme of animal experiments (Red arrows: 1.5 mg TNBS/50% EtOH enema dose; Orange arrows: 2.0 mg TNBS/50% EtOH enema dose; Grey arrows: 2.5 mg TNBS/50% EtOH enema dose). **B** Representative images of the colon with or without D-CeO_2_ treatment and **C** corresponding colon length. **D** Body weight change. **E** Spleen weight and organ index. **F** The endoscopic, **G** H&E staining and **H** IL-1β, TNF-α mRNA levels were used to assess the levels of inflammation. **I** Masson staining showing the collagen deposition in colonic tissue. **J** Immunofluorescence of colonic tissue with α-SMA. **K** TGF-β, Collagen 3, and α-SMA mRNA levels were illustrated by qRT-PCR analysis. One-way of ANOVA were performed for statistical comparison, *p < 0.05, **p < 0.01, ***p < 0.001

Encouraged by the satisfactory effects in vitro and good biocompatibility, D-CeO_2_ was further investigated for its therapeutic effects in TNBS-induced colitis, which is a commonly utilized animal model that shares significant properties with human Crohn's disease. The whole experimental procedure is illustrated in Fig. 7A. Healthy mice were kept for one week and divided into four groups. A chronic colitis model was established by weekly enemas with incremental amounts of TNBS. Mice were given D-CeO_2_ (30 mg/kg) every 2 days during the modeling process. D-CeO_2_ was delivered by hydrogel, thereby protecting its transit in the gastrointestinal tract.

After the experiment, executed mice by cervical dislocation. It was observed that the colon of mice in the TNBS group was significantly shorter than the control group, while the colon length of the TNBS + D-CeO_2_ group almost returned to normal (Fig. 7B, C). The body weight of mice in the TNBS and TNBS + D-CeO_2_ groups showed different degrees of weight loss after the first three enemas. However, the weight change of the TNBS + D-CeO_2_ group became lighter after the fifth week with the increasing number of enemas (Fig. 7D). The spleen weight and organ index of each group were also counted. As depicted in Fig. 7E, the TNBS group mice had increased spleen weight and higher liver and spleen organ index than the control group. In contrast, the values in the D-CeO_2_ group were decreased, indicating the reduction of inflammation. To more visually assess the colonic changes in each group during the modeling process, we examined the colons of the mice by endoscopy at the third, fifth, and seventh weeks of modeling. It was observed that the intestinal lumen was structurally stiff, with apparent ulcerative lesions in the TNBS group. In contrast, the other groups had red and moist intestinal mucosa with intact structure (Fig. 7F). H&E results showed that the TNBS-induced colitis mice had severe structural destruction of colonic tissues, which was improved by D-CeO_2_ treatment (Fig. 7G). The levels of several inflammation cytokines in colonic tissues were detected by qRT-PCR. As shown in Fig. 7H, IL-1β and TNF-α were significantly reduced after D-CeO_2_ application. These results powerfully demonstrated that D-CeO_2_ exhibited excellent anti-inflammatory ability in vivo.

After that, we further investigated the anti-fibrotic effects of D-CeO_2_ in vivo. Masson staining indicated fibrin deposition of the submucosal was significantly accumulated in the TNBS group. However, the TNBS + D-CeO_2_ group was not observed this lesion in colonic tissues (Fig. 7J). As shown in Fig. 7J, the immunofluorescence staining of frozen sections revealed that the expression of α-SMA, a marker protein of fibrosis, was dramatically increased in the TNBS group while decreased sharply in the TNBS + D-CeO_2_ group. The quantification of fluorescence intensity of α-SMA was shown in Additional file 1: Fig. S9. The results of qRT-PCR also verified that D-CeO_2_ could effectively reduce the mRNA levels of TGF-β1, Collagen 3, and α-SMA, thus exerting excellent anti-fibrotic effects in vivo (Fig. 7K).

### The therapeutic effect of D-CeO_2_ on DSS-induced chronic colitis

**Fig. 8:**
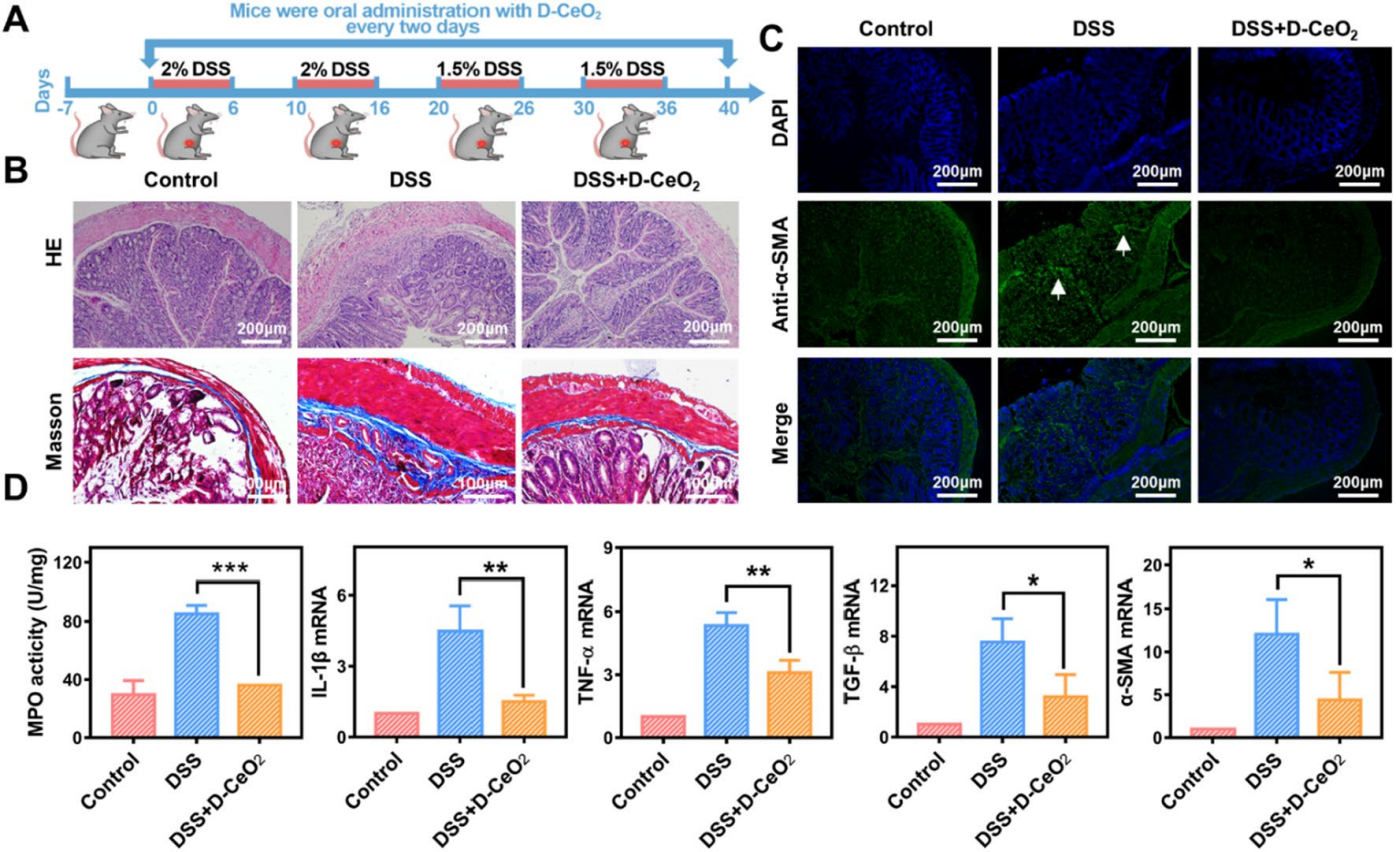
The anti-inflammatory and anti-fibrotic effects of D-CeO_2_ in DSS-induced chronic colitis. **A** The scheme of animal experiments. **B** H&E staining, Masson staining, and **C** immunofluorescence were used to assess the levels of inflammation and fibrosis. **D** The MPO activity and relative mRNA levels of IL-1β, TNF-α, TGF-β, and α-SMA in colonic tissues. One-way of ANOVA were performed for statistical comparison, *p < 0.05, **p < 0.01, ***p < 0.001

Next, we investigated the therapeutic ability of D-CeO_2_ in the DSS-induced chronic colitis model. As presented in Fig. 8A, the DSS-induced chronic colitis model was established by administering four cycles of DSS solution (1.5–2%) to mice. During the modeling process, mice were given D-CeO_2_ (30 mg/kg in hydrogel) every two days.

Similarly, Vital organs were collected and weighed. DSS group mice had increased spleen weight and higher liver and spleen organ index than the control group (Additional file 1: Fig. S6), indicating hepatosplenomegaly. In the D-CeO_2_ treatment group, it reduced slightly, confirming the reduction of inflammation in mice. H&E staining and Masson staining suggested that the colon microstructure was severely broken, and the fibrin deposition between the submucosa and the muscle layer was thickened in the DSS group. However, these lesions were significantly lighter in the DSS + D-CeO_2_ group (Fig. 8B). Immunofluorescence staining of α-SMA displayed an accumulation expression in the colonic tissues of the DSS group. Satisfactorily, it was not observed in the D-CeO_2_ treatment group (Fig. 8C). The quantification of fluorescence intensity of α-SMA was shown in Additional file 1: Fig. S10C. qRT-PCR results also confirmed that D-CeO_2_ significantly reduced mRNA levels associated with inflammation and fibrosis (Fig. 8D). These results indicated that D-CeO_2_ has anti-inflammatory and anti-fibrotic effects in the therapy of experimental colitis models, promising applications in alleviating IBD.

## Discussion

IBD is a chronic refractory disease in which intestinal fibrosis is a common complication. However, the effects of conventional treatments are limited, and as the disease progresses, intestinal fibrosis becomes an important factor in forcing patients to undergo surgical treatment [46, 47]. Previous studies have found both therapeutic and excellent CT imaging of CeO_2_ nanoparticles in experimental colitis models, which can alleviate the inflammatory state by scavenging ROS [32, 48]. Nevertheless, there are no relevant studies on the anti-fibrotic effects of CeO_2_ nanoparticles in IBD. Therefore, we investigated the applications of CeO_2_ nanoparticles with multi-ROS scavenging ability in the diagnosis as well as anti-inflammatory and anti-fibrotic treatment of IBD.

This study synthesized CeO_2_ and dextran-coated CeO_2_ (D-CeO_2_) nanoparticles. Under the same synthesis conditions, D-CeO_2_ had a smaller particle size than CeO_2_. This might be because the dextran molecules wrapped around the surface of nanoparticles during forming cerium oxide nanocrystals, preventing their further growth. The smaller the particle size of cerium oxide, the more oxygen defects and the better ROS scavenging ability [29]. Thus, we chose D-CeO_2_ as the future research object. The results confirmed that D-CeO_2_ exhibited significant SOD-mimicking and CAT-mimicking activity and had the capability of CT imaging. Moreover, the superior enzymatic activity could help reduce the damage caused by X-ray radiation during CT imaging compared to conventional contrast agents. As a high-performance enzyme, D-CeO_2_ exhibited good biosafety and efficient ROS scavenging ability at the cellular level. Notably, it also exerted anti-inflammatory and anti-fibrotic effects. The results at the animal level further suggested that D-CeO_2_ showed excellent efficacy in treating IBD by significantly downregulating the levels of pro-inflammatory and fibrosis-related cytokines.

Imperfectly, our study has not thoroughly investigated the specific molecular mechanism of D-CeO_2_ anti-fibrosis. It has been reported that ROS can influence the development of fibrosis mediated by activation of TGF-β1, which in turn can promote ROS production, inhibit antioxidant enzyme activity and lead to redox imbalance. This ultimately creates a vicious cycle and promotes the development of fibrosis-related diseases [49–51]. The inhibition of fibrosis in the heart, liver, lung, and kidney by reducing ROS has also been studied [52–55]. Moreover, it has been shown that CeO_2_ can influence the process of liver fibrosis by regulating TGF-β-related signaling pathways [56]. Therefore, combined with our results, we speculate that the molecular mechanism of D-CeO_2_ anti-fibrotic may be accomplished by scavenging ROS and thus regulating TGF-β-related signaling pathways. It is worth noting that although TGF-β is an important mediator of fibrogenesis activation since it is also an important cytokine associated with inflammation, further studies are needed to demonstrate whether anti-fibrosis by blocking TGF-β may exacerbate inflammation or have other adverse effects.

## Conclusions

In summary, we developed D-CeO_2_ nanozymes with the ability of ROS scavenging as well as CT imaging, which has the potential to realize the integration of IBD diagnosis and therapy. Notably, our work provided a new solution for the treatment and prognosis of IBD. In addition to alleviating the inflammatory state of IBD, D-CeO_2_ also had a regulatory role in preventing the onset and progression of fibrosis. Without appreciable systemic toxicity, D-CeO_2_ held the promise of integrated applications for diagnosis and therapy, pioneering the exploration of nanozymes with ROS scavenging capacity in the anti-fibrotic treatment of IBD.

## Methods

### Materials

Cerium nitrate (Ce(NO_3_)_3_·6H_2_O) was obtained from Sinopharm Group Chemical Reagents Co., Ltd; Ammonia (NH_3_·H_2_O, 30%) was purchased from Shanghai Macklin Biochemical Technology Company; Dextran T-10 was obtained from Xi’an Ruixi Biological Technology Co., Ltd; 5,5-dimethyl-1-pyrroline N-oxide (DMPO), L-methionine (L-met), nitrogen blue tetrazolium (NBT), and riboflavin were purchased from Aladdin Industrial Inc. Hydrogen peroxide (H_2_O_2_, 30%) was obtained from Tianjin Damao Chemical Reagent Co., Ltd; 2,7-dichlorofluorescein diacetate (DCFH-DA) was purchased from Beyotime Biotechnology Co., Ltd.

### Synthesis of cerium oxide (CeO_2_) and dextran-coated cerium oxide (D-CeO_2_)

Cerium oxide (CeO_2_) was synthesized by the precipitation method. Briefly, 1 mL of 1 M Ce(NO_3_)_3_·6H_2_O was added to the 6 mL of 30% ammonia drop by drop and stirred for 24 h. Finally, the solution was alternately washed with deionized water and ethanol three times at 9000 rpm for 5 min each. D-CeO_2_ were synthesized by precipitation based on the previously reported protocol [31, 32]. Briefly, 1 mL of 1 M Ce(NO_3_)_3_·6H_2_O was added dropwise to 2 mL of 0.2 M Dextran T-10 solution. Then, this mixture was added to 30% ammonia (6 mL) and stirred for 24 h, the color of the solution could be observed to change from yellow to dark brown gradually. The suspension obtained was first centrifuged twice to extract the larger precipitates, then transferred to an ultrafiltration tube (MWCO 100 kDa) and centrifuged three times at 4500*g* for 15 min each.

### Preparation of chitosan/alginate hydrogel

Chitosan/alginate hydrogel was prepared according to our reported protocol [57, 58]. Firstly, dissolved chitosan with an appropriate amount of acetic acid, then adjusted the pH of the solution to 7 and ensured that the ultimate concentration is 0.6% (wt/vol). The sodium alginate solution (1.4%) was prepared by dissolving the sodium alginate in 0.15 mol/L NaCl and stirring overnight. The polysaccharide solution was made by mixing the chitosan solution with the sodium alginate in a 1:1 ratio. The chelating solution was made by mixing 30 mM Na_2_SO_4_ and 70 mM CaCl_2_ in a 1:1 ratio. Finally, the hydrogel was prepared by mixing the polysaccharide and the chelating solution in a 2:1 volume. D-CeO_2_ was dissolved in the polysaccharide solution before gavage. For gavage, mice were given 100 μL of polysaccharide solution followed by 50 μL of chelated solution.

### Characterization

The morphology of the synthesized CeO_2_ and D-CeO_2_ was characterized using transmission electron microscopy (TEM, JEM-2100). The hydrodynamic particle size and zeta potential were taken with a Malvern laser particle size analyzer (Malvern Instruments, UK). Fourier Transform infrared spectroscopy (FT-IR, Thermo Fisher Nicolet 5700) was performed to observe the characteristic peaks of CeO_2_, D-CeO_2,_ and Dextran. X-ray diffraction (XRD, Bruker D8 Advance) and X-ray photoelectron spectroscopy (XPS, Thermo Fisher ESCALAB Xi+) were determined by the Analytical Testing Centre of Xi’an Jiaotong University.

### The SOD-mimicking activity of D-CeO_2_

The SOD-mimicking activity was detected by electron spin resonance (ESR) and nitrogen blue tetrazolium (NBT) assays. Under light conditions, L-methionine (L-met) and riboflavin can react to produce superoxide anion (·O_2_^−^), and the DMPO can capture ·O_2_^−^. 50 µL of PBS (25 mM, pH 7.4),10 µL of L-met (130 mM), riboflavin (200 µM), EDTA (100 µM), and DMPO (250 mM) were added sequentially to the reaction system. Then added 10 µL of D-CeO_2_ (100 μg/mL) to the above reaction system. After 20 min of light, analysis was performed by Bruker A300-9.5/12 spectrometer.

In the presence of L-Met and riboflavin, NBT undergoes a photochemical reduction reaction to produce blue methyl hydrazone after illumination, which has the maximum absorption at 560 nm. SOD enzyme can inhibit the reduction of NBT under light. PBS, riboflavin, L-Met, and EDTA were added sequentially in the reaction plate, then added 10 µL of NBT (750 μM) and 10 µL of D-CeO_2_ nanoparticles with different concentrations. After illumination for 10 min, the absorption value at 560 nm was measured by enzyme standard (Bio Tek NEO2), and SOD-mimicking activity was calculated.

### The CAT-mimicking activity of D-CeO_2_

The CAT-mimicking activity of D-CeO_2_ nanoparticles was assessed by analyzing the amount of O_2_ generation from the catalytic decomposition of H_2_O_2_ using the dissolved oxygen electrode. Briefly, H_2_O_2_ and different concentrations of D-CeO_2_ nanoparticles were added to the reaction bottles, then dissolved oxygen generated during 900 s was recorded with 30 s intervals. The final concentration of H_2_O_2_ was maintained at 120 mM.

### Cell culture

Raw 264.7 and NIH 3T3 cells were cultured in DMEM medium (Gibco), and Colon-26 cells were cultured in RPMI Medium 1640 (Gibco) medium. The complete medium contains 10% fetal bovine serum and 1% penicillin/streptomycin. All the cells were grown at 37 °C in 5% CO_2_ humidified incubator.

### Cellular uptake

Raw 264.7, Colon-26, and NIH 3T3 cells were chosen to perform the cellular uptake assay. Firstly, DiL fluorescent dye was added during the synthesis of D-CeO_2_ to make it monitorable under fluorescence microscopy. Then cells were incubated with D-CeO_2_ for 6 h and performed by fluorescent microscopy to observe the cellular uptake.

### Cell viability assay

Raw 264.7 cells and Colon-26 cells were seeded in cell culture plates, respectively. After cell adherence, different concentrations of nanoparticles were added and co-incubated with cells for 24 h and 48 h. Then the culture was terminated, and MTT was incubated for 4 h. Aspirated medium and added 150 μL DMSO to each well. The absorbance at 490 nm was detected by enzyme standard, and cell viability was calculated.

### Intracellular ROS scavenging ability of D-CeO_2_

The ability of D-CeO_2_ to scavenge ROS was assayed using DCFH-DA. Firstly, different concentrations of D-CeO_2_ (100, 500 μg/mL) were used to treat Raw 264.7 cells for 6 h. Next, a fresh medium containing 1 mM H_2_O_2_ was used to stimulate all groups for 3 h except the negative group. After washing with PBS, DCFH-DA (10 μM) was added and co-incubated with cells at 37 °C for 1 h. Then intracellular fluorescence was observed by fluorescence microscopy. Alternatively, the cells were washed with PBS and collected for detection by flow cytometry.

### The protective ability of D-CeO_2_ against oxidative stress damage

We selected Raw 264.7 and Colon-26 cells to investigate the protection of D-CeO_2_ against H_2_O_2_-induced oxidative stress damage. Briefly, cells were seeded to each well of 96-well plates. After 12 h, added 1 mM H_2_O_2_ to induce the cells for 1 h in addition to the negative control group. The D-CeO_2_ treatment group was incubated with H_2_O_2_ in conjunction with different concentrations of nanoparticles (100, 200, 500 μg/mL). The cells were then washed with PBS and further cultured in a complete medium for 24 h. MTT assay was used to measure cell viability.

### In vitro anti-inflammatory assay

Firstly, Raw 264.7 cells were seed into a 6-well plate at 1 × 10^4^ cells/well. After 12 h, washed cells three times with PBS and then incubated with D-CeO_2_ nanoparticles dispersed in completed media for 6 h. The final concentration of D-CeO_2_ was 500 μg/mL. Lipopolysaccharide (LPS) had a final concentration of 100 ng/mL was used to stimulate cells for 24 h. Cells were collected for RNA analysis by centrifugation at 2000 rpm for 3 min.

### Scratch test

Firstly, pre-adhered the scratch inserts to the bottom of 12-well plates. Next, NIH 3T3 cells were plated uniformly into the cell culture plate. After cells reached the monolayer state, the scratch insert was removed. Suspended cells were washed with PBS. Next serum-free medium containing 10 ng/mL TGF-β1 and different concentrations of material was added to all groups except the negative group. Cells were future-cultured for 24 h and then observed by using a microscope.

### Immunofluorescence staining

NIH 3T3 cells were seeded in cell crawling sheets and incubated with D-CeO_2_ for 6 h. Then added TGF-β1 (10 ng/mL) to each well. After 24 h stimulating, 4% paraformaldehyde was used to fix cells for 15 min, 0.1% TritonX-100 was used to permeabilize for 10 min, and 2% BSA was used to bloke for 60 min. For staining of α-SMA (14395-1-AP, Proteintech) and Collagen 1 (14695-1-AP, Proteintech), the cells were incubated with primary antibody at 4 °C overnight. The next day, cells were incubated with fluorescent secondary antibodies (A1108, Invitrogen) for 60 min at 37 °C, followed by washing for 10 min three times. Images were captured by fluorescence microscopy.

### RNA extraction and qRT-PCR

According to the instructions, total RNA was isolated from cells or tissues using the Total RNA Extraction Kit (R0027, Beyotime). The total RNA concentration was determined by NanoDrop spectrophotometer. 1.0 μg of isolated RNA was used to prepare cDNA with cDNA Synthesis Premix (D7185M, Beyotime). qRT-PCR was performed on a BIOER Quant Gene 9600 real-time PCR system using Green Master (Roche) with 20 μL reaction mixture. The primer sequences were presented in Additional file 1: Table S1. Relative mRNA levels were quantified by the 2^−ΔΔCt^ method.

### Western blot

NIH 3T3 cells were treated as described previously. At the end of incubation, cells were collected and lysed in RIPA buffer containing protease and phosphatase inhibitor cocktail. The concentration of protein was assessed by NanoDrop spectrophotometer. Then loaded the protein (200 μg) on SDS-PAGE (10% or 12%) and blotted onto NC membranes. After blocking, the primary antibodies were incubated with membranes overnight at 4 °C to detect the specific protein. Anti-α-SMA (14395-1-AP, Proteintech), anti-Collagen 1 (14695-1-AP, Proteintech), and anti-GADPH (GB12002, Servicebio) antibodies used in this part were configured at a concentration of 1:1000. The HRP-conjugated secondary antibodies against mouse (1:3000, GB23301, Servicebio) or HRP-conjugated secondary antibodies against rabbit (1:3000, GB23303, Servicebio) were used to detected appropriate primary antibodies. Bands were visualized with ECL-system, and images were captured using the chemiluminescence instrument.

### Animals

Female BALB/c mice were purchased from Xi'an Keao Biotechnology Co., Ltd. Female C57BL/6 mice were obtained from the Experimental Animal Center of Xi'an Jiaotong University. The animals were kept under 22–25 °C, 65 ± 5% humidity with a 12 h light–dark cycle, and fed regular and free drinking water. All experiments complied with the Institutional Animal Care and Use Committee at Xi’an Jiaotong University.

### CT imaging

Since CeO_2_ is the main component of D-CeO_2_ that exerts a CT imaging effect, we quantified the Ce content in D-CeO_2_ by ICP-MS and calculated the CeO_2_ content in it. The D-CeO_2_ concentrations covered in this section are representative of the CeO_2_ concentrations. Iohexol (an FDA-approved CT contrast agent) was used as a control to study the CT imaging properties of D-CeO_2_. Firstly, different concentrations of Iohexol solution (0.55, 1.1, 2.25, 4.5, 9.0, 18.0 mg/mL) and D-CeO_2_ solution (0.6, 1.2, 2.4, 4.7.9.4, 18.9 mg/mL) were measured in vitro to compare their CT imaging ability. Next, a DSS-induced acute colitis mouse model was established to explore the CT imaging ability of D-CeO_2_ in vivo*,* referencing the previous literature [59]. Briefly, C57BL/6 mice were given water containing 2% (w/v) DSS for 7 consecutive days. The healthy group was given the same DSS-free drinking water. Then mice were gavaged with iohexol or D-CeO_2_ at a dose of 38 mg/kg. After administration, in vivo CT imaging was performed at 5 min, 30 min, 60 min, 120 min, and 24 h.

### In vivo biocompatibility evaluation

To evaluate the biocompatibility of D-CeO_2_ in vivo, C57BL/6 mice were orally administered D-CeO_2_ (30 mg/kg in hydrogel) for 7 consecutive days and sacrificed one month later. At the end of the experiment, vital organs and the gastrointestinal tract were taken for H&E staining. Blood samples were analyzed using a hematology analyzer and compared with the control group.

### TNBS-induced chronic colitis mouse model

Six to eight-week-old female BALB/c mice, weighing 18–20 g, were used to establish a TNBS-induced chronic colitis model reference to previous studies [59–61]. The specific experimental steps were as follows: Firstly, randomly divide the mice into 4 groups (Control, EtOH, TNBS, TNBS + D-CeO_2_) and fasted overnight before each enema. The enema needle was inserted into the anus for 4 cm to inject 100 μL of enema solution after mice were anesthetized with isoflurane. Then mice were immediately inverted for 1–2 min to ensure that the solution was retained in the entire colon. The control group was fed a normal diet without special treatment, the EtOH group was administered 50% ethanol solution via enema, and the TNBS group and TNBS + D-CeO_2_ group were given increasing concentrations of TNBS via enema once per week for 7 weeks consecutively (1.5 mg/0.1 mL for 1st and 2nd weeks, 2.0 mg/0.1 mL for 3rd and 4th weeks, and 2.5 mg/0.1 mL for the last three weeks). Mice in the TNBS + D-CeO_2_ group were orally administrated D-CeO_2_ (30 mg/kg in hydrogel) every 2 days in addition to weekly TNBS. Mice were executed after 2 days of the last enema. The body weight was recorded during the experimental period, and the intestinal condition was observed through a multifunctional small animal soft endoscope (MiniScope 2V, SHINOVA) on the day after the enema. After mice were sacrificed, recorded the colon length and collected colon tissues for RNA analysis, histopathological analysis, and immunofluorescent staining.

### DSS-induced chronic colitis mouse model

Female C57BL/6 mice aged 6–8 weeks, weighing 18–20 g, were used to set up a DSS-induced chronic colitis model according to the previous study [62]. First, we divided the mice into 3 groups (Control, DSS, DSS + D-CeO_2_). The mice were given 1.5–2% DSS in drinking water for 4 cycles except for the control group. On days 1–6 and 11–16, mice received drinking water containing 2% DSS. On days 21–26 and 31–36, mice received drinking water containing 1.5% DSS. On the remaining days, mice received regular water. Mice in the DSS + D-CeO_2_ group were orally administrated D-CeO_2_ (30 mg/kg in hydrogel) every two days starting from day 0. After mice were sacrificed, the colon lengths were measured and recorded, and colonic tissues were collected for analysis.

### Histological analysis

Colon tissues were histologically analyzed by hematoxylin, eosin staining (H&E), and Masson staining. Briefly, 4% paraformaldehyde was used to fix the obtained colonic tissue, followed by paraffin embedding and cutting to 5 μm layer thickness. The sections were placed on slides for drying and subsequent staining. Masson staining was performed using a kit (G1346, Solarbio) according to the instructions.

### Immunofluorescence assay of the colon tissues

Colon tissue was embedded with OCT, cut into 4–6 μm sections. After permeabilizing and blocking, they were incubated with primary antibodies overnight at 4 °C, fluorescent secondary antibodies at 37 °C, and cell nuclei were stained with DAPI. Fluorescence microscopy was used to capture images.

### MPO activity assay

MPO activity was analyzed by colorimetric method, and the MPO assay kit (A044-1-1, Nanjing Jiancheng Institute of Biological Engineering) was used to quantify MPO activity according to the manufacturing instructions.

### Statistical analysis

GraphPad Prism 7 software was used for statistical analysis. One-way of variance (ANOVA) and t-tests were performed for statistical comparison. Statistically significant was indicated as *p < 0.05, **p < 0.01, ***p < 0.001.

## Supplementary Information

**Additional file 1: Figure S1.** UV−vis spectrum of D-CeO_2_. **Figure S2. A** Dependence between the ·O_2_^–^ elimination efficiency of D-CeO_2_ and SOD. **B** Dependence between the oxygen production velocities in the initial 5 min of D-CeO_2_ and CAT. SOD-mimicking and CAT-mimicking activity after incubated at **C** different temperatures (4, 25, 37 °C) and **D** various pH values (1.5, 6.0, 7.4, 8.0) for 4 h. **Figure S3.** Fluorescence image of Raw 264.7, Colon-26, and NIH 3T3 cells incubated with D-CeO_2_ for 6 h. **Figure S4.** Fluorescent images of calcein-AM/PI co-stained Raw 264.7, Colon-26, and NIH 3T3 cells after various treatments. **Figure S5.** Quantification of fluorescence intensity of **A** α-SMA and **B** Collagen 1. **Figure S6**. **A** Spleen weight and **B** organ index of DSS-induced model. **Table S1.** Sequences of the primers used for qRT-PCR.

## Data Availability

All data generated or analysed during this study are included in this published article and its supplementary information files. The datasets used or analyzed during the study are available upon reasonable request.

## Abbreviations

IBD: Inflammatory bowel disease
ROS: Reactive oxygen species
CAT: Catalase
SOD: Superoxide dismutase
D-CeO_2_: Dextran-coated cerium oxide nanozymes
DCFH-DA: 2′,7′-Dichlorodihydrofluorescein diacetate
H_2_O_2_: Hydrogen peroxide
LPS: Lipopolysaccharide
H&E: Hematoxylin and eosin
TEM: Transmission electron microscopy
XRD: X-ray powder diffraction
XPS: X-ray photoelectron spectra
ESR: Electron spin resonance spectroscopy
ICP–MS: Inductively coupled plasma–mass spectrometry
MPO: Myeloperoxidase
qRT-PCR: Quantitative real-time polymerase chain reaction
TNBS: 2,4,6-Trinitrobenzene sulfonic acid
