# Supplementary material for: Orally administration of cerium oxide nanozyme for computed tomography imaging and anti-inflammatory/anti-fibrotic therapy of inflammatory bowel disease
Source: J Nanobiotechnology. 2023 Jan 19;21:21. doi: 10.1186/s12951-023-01770-0 (PMC9854161; doi:10.1186/s12951-023-01770-0)
Supplement: Supplementary file 1 — Additional file 1: Figure S1. UV−vis spectrum of D-CeO2. Figure S2. A Dependence between the ·O2– elimination efficiency of D-CeO2 and SOD. B Dependence between the oxygen production velocities in the initial 5 min of D-CeO2 and CAT. SOD-mimicking and CAT-mimicking activity after incubated at C different temperatures (4, 25, 37 °C) and D various pH values (1.5, 6.0, 7.4, 8.0) for 4 h. Figure S3. Fluorescence image of Raw 264.7, Colon-26, and NIH 3T3 cells incubated with D-CeO2 for 6 h. Figure S4. Fluorescent images of calcein-AM/PI co-stained Raw 264.7, Colon-26, and NIH 3T3 cells after various treatments. Figure S5. Quantification of fluorescence intensity of A α-SMA and B Collagen 1. Figure S6. A Spleen weight and B organ index of DSS-induced model. Table S1. Sequences of the primers used for qRT-PCR. [file 12951_2023_1770_MOESM1_ESM.docx]

**Additional file 1**

**Orally Administration of Cerium Oxide Nanozyme for Computed Tomography Imaging and Anti-Inflammatory/Anti-Fibrotic Therapy of Inflammatory Bowel Disease**

Yameng Cao^1,2,5^, Kai Cheng^3^, Mei Yang^1,2,5^, Zhichao Deng^1,2,5^, Yana Ma^1,2,5^, Xiangji Yan^1,2,5^, Yuanyuan Zhang^1,2,5^, Zhenzhen Jia^1,2,5^, Jun Wang^4^, Kangsheng Tu^1*^, Jie Liang^6*^, and Mingzhen Zhang^1,2,5*^

Affiliations:

1. Department of Hepatobiliary Surgery, the First Affiliated Hospital of Xi'an Jiaotong University, Xi'an, Shaanxi, 710061, China
2. School of Basic Medical Sciences, Xi'an Key Laboratory of Immune Related Diseases, Xi'an Jiaotong University, Xi'an, Shaanxi, 710061, China
3. Britton Chance Center for Biomedical Photonics at Wuhan National Laboratory for Optoelectronics-Hubei Bioinformatics & Molecular Imaging Key Laboratory, Department of Biomedical Engineering, College of Life Science and Technology, Huazhong University of Science and Technology, Wuhan, Hubei, 430074, China
4. Department of Emergency and Critical Care Medicine, the First Affiliated Hospital of Xi’an Jiaotong University, Xi’an 710061, China
5. Key Laboratory of Environment and Genes Related to Diseases, Xi'an Jiaotong University, Ministry of Education, Xi'an, Shaanxi, 710061, China
6. Xijing Hospital of Digestive Diseases, Air Force Military Medical University, Xi'an, Shaanxi, 710068, China


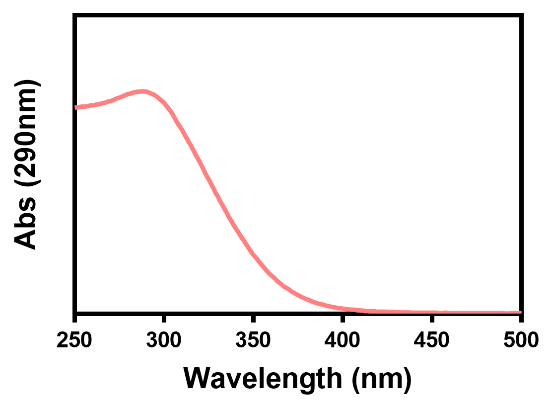


**Fig. S1.** UV−vis spectrum of D-CeO_2_.


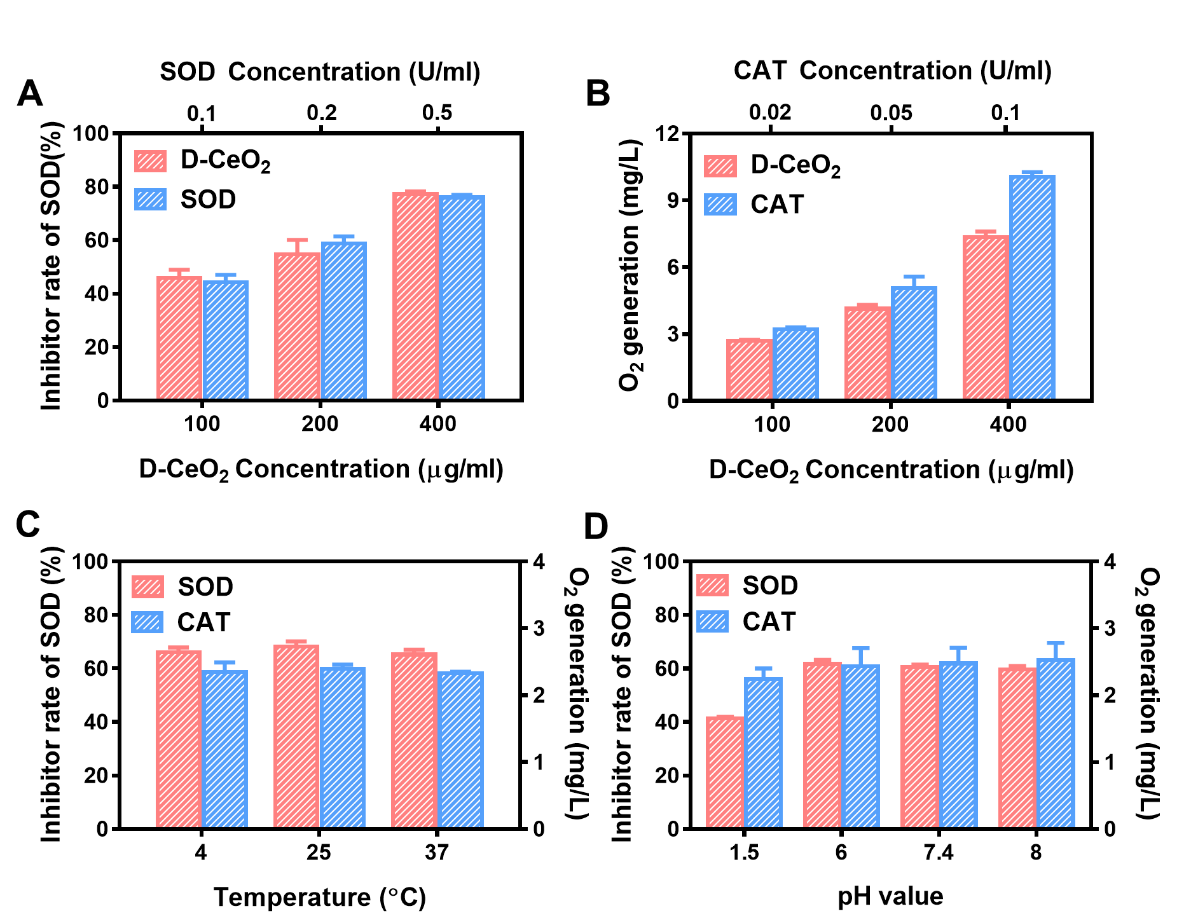


**Fig. S2. (A)** Dependence between the •O_2_^–^ elimination efficiency of D-CeO_2_ and SOD. **(B)** Dependence between the oxygen production velocities in the initial 5 min of D-CeO_2_ and CAT. SOD-mimicking and CAT-mimicking activity after incubated at **(C)** different temperatures (4, 25, 37 ^◦^C) and **(D)** various pH values (1.5, 6.0, 7.4, 8.0) for 4 h.


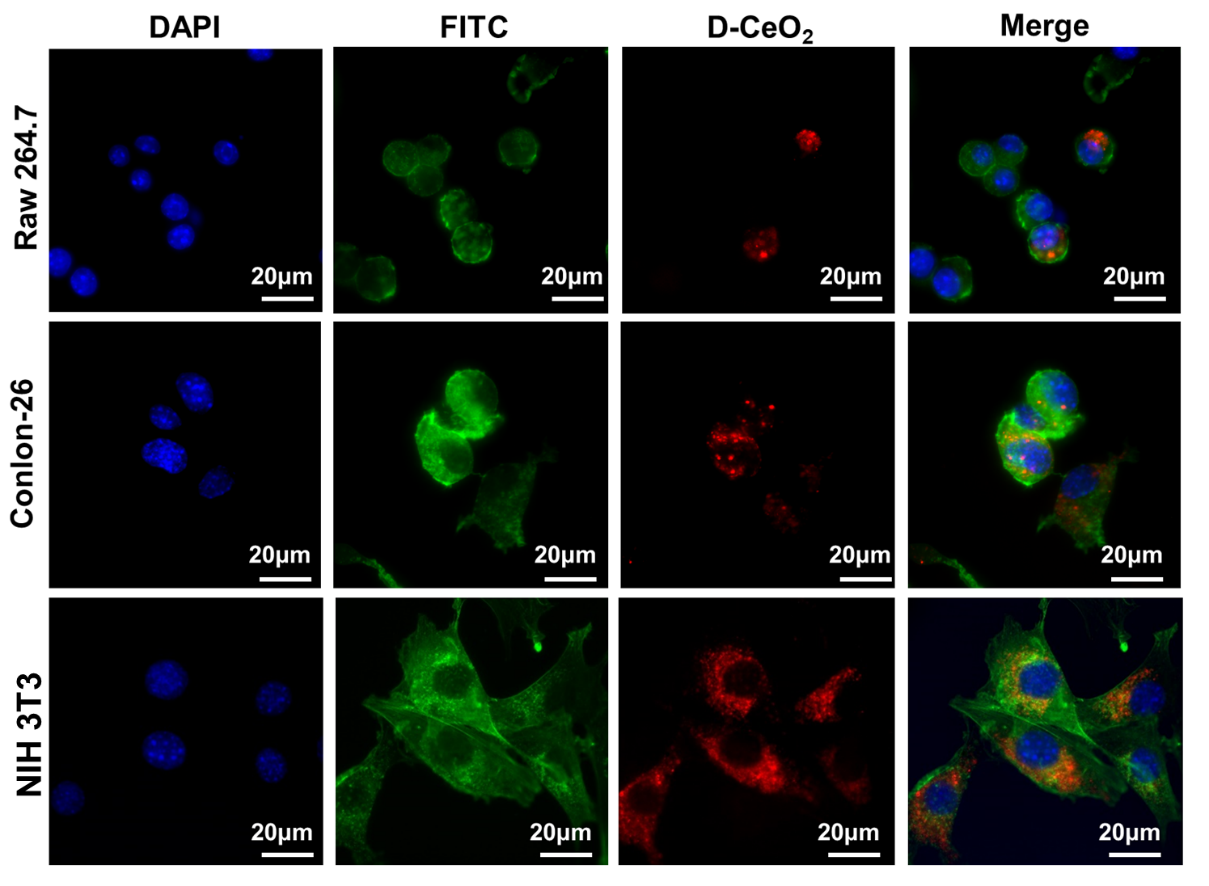


**Fig. S3.** Fluorescence image of Raw 264.7, Colon-26, and NIH 3T3 cells incubated with D-CeO_2_ for 6 h.


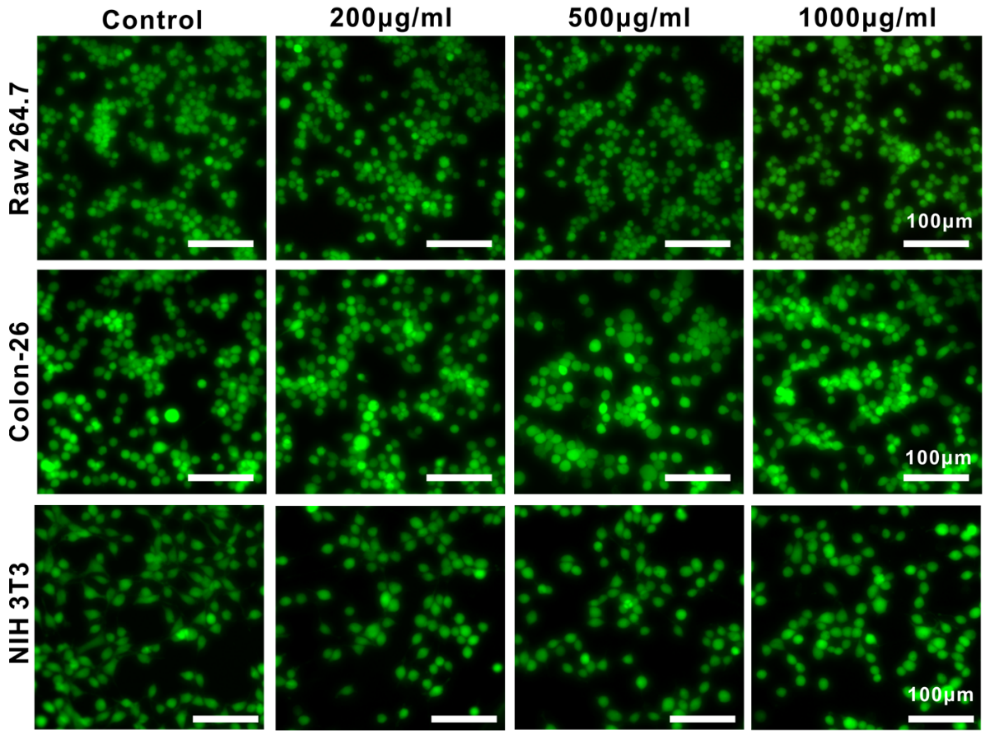


**Fig. S4.** Fluorescent images of calcein-AM/PI co-stained Raw 264.7, Colon-26, and NIH 3T3 cells after various treatments.


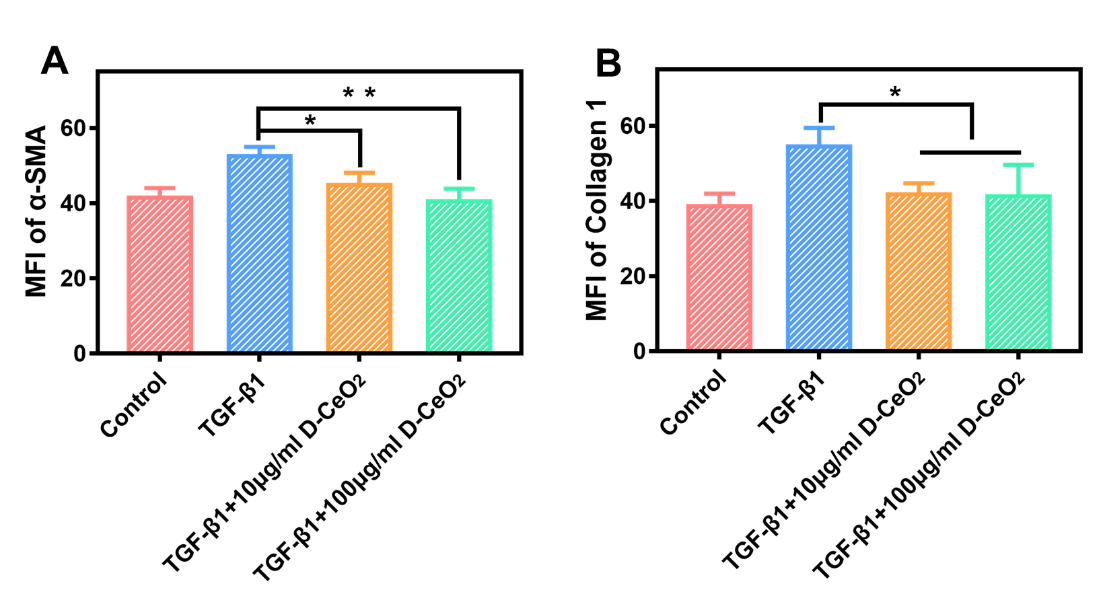


**Fig. S5.** Quantification of fluorescence intensity of **(A)** α-SMA and **(B)** Collagen 1.


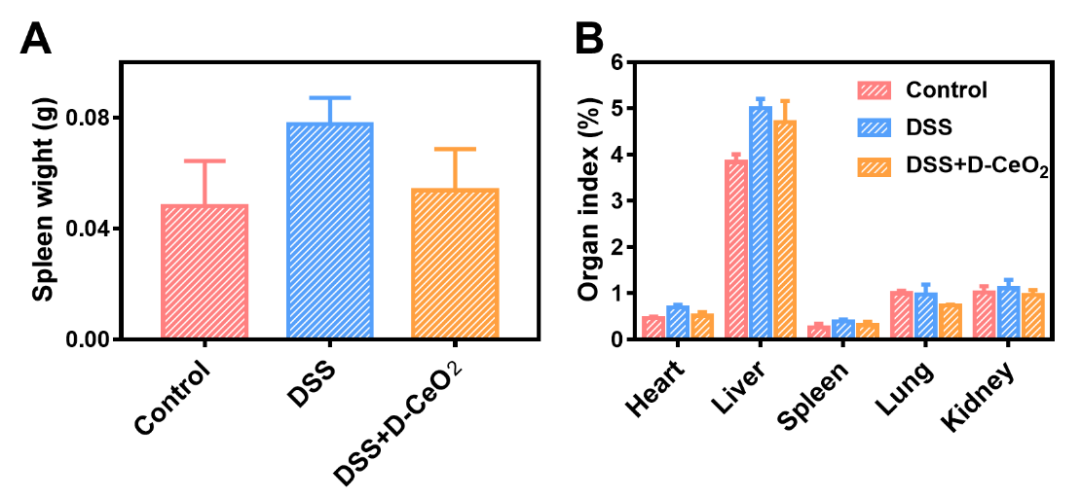


**Fig. S6**. (**A**) Spleen weight and (**B**) organ index of DSS-induced model.

| Primer name | Sequences (5’-3’) |
| --- | --- |
| IL-1β | F: TCGCTCAGGGTCACAAGAAA |
|  | R: CATCAGAGGCAAGGAGGAAAAC |
| TNF-α | F: AGGCTGCCCCGACTACGT |
|  | R: GACTTTCTCCTGGTATGAGATAGCAAA |
| IL-6 | F: ACAAGTCGGAGGCTTAATTACACAT |
|  | R: TTGCCATTGCACAACTCTTTTC |
| iNOS | F: GTGGTGACAAGCACATTTGG |
|  | R: AAGGCCAAACACAGCATACC |
| TGF-β | F: GCAACAATTCCTGGCGTTAC |
|  | R: AGAGCAGTGAGCGCTGAATC |
| α-SMA | F: GGACGTACAACTGGTATTGTGC |
|  | R: TCGGCAGTAGTCACGAAGGA |
| Collagen 3 | F: TGACTGTCCCACGTAAGCAC |
|  | R: GCTGCACATCAACGACATCT |

**Table S1.** Sequences of the primers used for qRT-PCR
